# Mentorship as an overlooked dimension of research capacity strengthening: how to embed value-driven practices in global health

**DOI:** 10.1136/bmjgh-2023-014394

**Published:** 2024-01-04

**Authors:** Candice Bonaconsa, Vrinda Nampoothiri, Oluchi Mbamalu, Sipho Dlamini, Surya Surendran, Sanjeev K Singh, Raheelah Ahmad, Alison Holmes, Muneera A Rasheed, Marc Mendelson, Esmita Charani

**Affiliations:** 1 Division of Infectious Diseases and HIV Medicine, Department of Medicine, Groote Schuur Hospital, University of Cape Town, Cape Town, South Africa; 2 Department of Health Sciences Research, Amrita Institute of Medical Science, Amrita Vishwa Vidyapeetham, Kochi, Kerala, India; 3 Department of Health Systems and Equity, The George Institute for Global Health, Hyderabad, India; 4 Department of Infection Control and Epidemiology, Amrita Institute of Medical Science, Amrita Vishwa Vidyapeetham, Kochi, Kerala, India; 5 School of Health Sciences City, University of London, London, UK; 6 Faculty of Health and Life Sciences, University of Liverpool, Liverpool, UK; 7 Centre for International Health, Department of Global Public Health and Primary Care, University of Bergen, Bergen, Norway

**Keywords:** Public Health, Review, Global Health

## Abstract

Mentorship in global health remains an overlooked dimension of research partnerships. Commitment to effective mentorship models requires value-driven approaches. This includes having an understanding of (1) what mentorship means across different cultural and hierarchical boundaries in the health research environment, and (2) addressing entrenched power asymmetries across different aspects including funding, leadership, data and outputs, and capacity strengthening. Existing guidance towards equity and sustainability fails to inform how to navigate complex relationships which hinder effective mentorship models. We focus this perspective piece on human capacity strengthening in research partnerships through mentorship. Using a case study of a research partnership, we describe the lessons learnt and the challenges faced in the mentor mentee relationship while maintaining an effective and sustainable partnership. Human capacity strengthening must research projects and collaborations, and recognise local leadership and ownership. To be transformative and effective, practices need to be driven by common values across research teams.

**Summary box**

- There is a shift to understanding the need for equitable global health partnerships and research collaborations.
- Mentorship is an important but overlooked dimension in research partnerships.
- We draw on our experiences of international research collaboration to define how mentorship can be sustainably developed as part of human capacity strengthening.
- Specifically focusing on the need to recognise the need for a value-driven approach to human capacity development in research partnerships.

## Introduction

Effective mentorship in research partnerships in global health is hindered by power asymmetries between and within countries.^1–4^ This is in part because global health funding, hence power, remains centred in institutions in high-income countries (HICs), even when attempting to address inequities in low-income and middle-income countries (LMICs).^5 6^ This results in little or no motivation to implement impactful mentorship practices. Although there has been extensive discussion about equity and its absence in global health,^7–10^ many questions still remain. For example, what does equity look like in practice? Can it ever be achieved or measured effectively when historical imbalances exist and when motivations for operating in the global health field are heavily influenced by geography, economics and politics? A gap exists in developing interventions and indicators that measure progress towards more equitable international research collaborations, including operating from a premise of shared power and decision-making to recognise, strengthen and celebrate local leadership in health research.^10–12^ Providing mentorship, particularly for early career researchers (ECRs), is critical to this end as a strategy to address these inequities. It is easier to learn new behaviours than unlearn old ones, and therefore focusing on ECRs regardless of geography maybe a more sustainable means to building better teams. Another aspect to consider is how to measure progress in capacity strengthening. Identifying indicators can provide a tool to researchers, collaborators, mentors and mentees to maximise the intended impact of their work and enable funders to assess and monitor the health of partnerships and the progress of potential and existing collaborations beyond publications.

A recent paper defined equitable partnerships in global health as ‘mutually beneficial and power-balanced partnerships and processes leading to equitable human and environmental health outcomes (which they refer to as “products”) on a global scale.’^13^ According to this definition, ensuring shared power and mutually beneficial relationships are at the core of global health equity. It is important to equip researchers embarking on collaborations with direction on how to assure balance of power to achieve the desired outcomes. Most funders and donors use capacity strengthening as a proxy for finding and achieving common goals in partnerships.^14–16^ The WHO criteria for research capacity strengthening in LMICs operate on seven principles of: networking and collaboration, understanding local contexts, ensuring local ownership, monitoring and evaluation, governance and leadership, strong supervision and mentorship, and planning for continuity.^16^ These criteria are collectively referred to as the ESSENCE criteria. This model works on the premise of power imbalances with funding and expertise from HIC and non-governmental organisations leading the change. In practice, this is far from the reality in many systems where there is a lot more learning and development that is bidirectional, with global health actors situated in HICs benefiting much more in the long-term through their capacity development than is written about.^10 17^ This absence of guidance on how those from better resourced countries can learn from those in less-resourced countries has been previously highlighted by Redman-MacLaren *et al*.^18^ There are long-lasting benefits to capacity strengthening, particularly in human capacity investment and strengthening. From this perspective, the role of mentorship is key, though it is often overlooked as a core dimension of equitable partnerships.

## Mentorship: the central pillar of capacity strengthening

Mentorship, especially of early ECRs, can provide opportunities for long-lasting change not only in outcomes of studies and career progression, but in shifting the culture of practice towards more equitable partnerships over time, specifically emphasising the significance of mentorship for ECRs in HICs. Similarly, a new path to practice is emerging in global health in response to a positive move to combat disparities and inequities in research across income settings recommending demand-driven and locally led research as well as mentorship.^1^ Therefore, embedding mentorship as part of capacity strengthening and as a meaningful strategy to nurture leadership in researchers should be a central objective in all research collaborations.

Mentorship is defined as a mutually beneficial partnership between the mentor and mentee where the complexity of context and culture must be considered. Reciprocity is a core component of this dynamic relationship that values individual development in addition to technical skills.^2^ To optimise individual development, it is important to focus on both technical skills, often referred to as hard skills, and soft skills, such as leadership and communication.^19^ Healthcare and research leaders frequently lack the necessary preparation and support when it comes to developing and using soft skills.^20^ Soft skills can enable resilient health system actors to lead in difficult and complex environments and should be a key consideration in mentorship.^21^

Recognising mentorship as a critical dimension of capacity strengthening takes it beyond the realm of short-term project -oriented goals, ensuring a long-lasting legacy of human capital where both members of the dyad benefit from the ongoing relationship. A mentorship model that is built on trust and respect with responsibilities for mentors and mentees can aid in creating willingness in mentees to be trained to take on an independent path.^3^ Support from funders, mentors, collaborating institutions and the motivation of mentees are key to success. We provide a framework based on our own experiences on how mentorship as part of human capacity strengthening can be achieved, monitored and sustained in multinational research partnerships. We provide an indicator checklist for integrating a mentorship model in partnerships across different dimensions, including funding and resource allocation, leadership, data equity, outputs management and planning. Paving a way for managing expectations across and within teams, we propose the development of Memorandum of Understanding (MoU) to provide a clear direction for researchers of different seniority working across teams to understand the expectations and responsibilities that are transparent and regularly reviewed and updated.

In the field of antimicrobial resistance (AMR) and antimicrobial stewardship (AMS), international collaboration is key to the scale-up of successful interventions and towards a global response to the threat of AMR.^22 23^ The most critical challenges in AMR are concentrated in LMICs as is the expertise to address these challenges. The drivers for making change however and much of the power still remain in a few select HICs.^5 6 24^ Thus, the concept of AMR is one that exemplifies the hierarchies and tensions between ‘global’ (ie, Western biomedical) and ‘local’ knowledge.^9^ To ensure equity within global health, intentional strategic efforts need to shift and share the power of decision-making that impacts which health interventions are implemented where and how resources are allocated.^25^ In order to achieve this, power asymmetries within all spaces of global health, including within academic institutes need to be addressed, with inclusion of capacity strengthening of ECRs regardless of geographic location. With this background and in response to the path to mentorship,^3 4 11^ we provide a visual framework towards equitable partnership and mentorship in global health, using a case study nested within a multinational research collaboration involving researchers and institutions in the UK, South Africa (SA) and India that aimed to understand the drivers for antibiotic prescribing and infection management across surgical pathways.^12 26–33^

The Antibiotic use across Surgical Pathways—Investigating, Redesigning and Evaluating Systems project (ASPIRES—https://www.imperial.ac.uk/arc/aspires/) funded by the UK ESRC prioritised north–south and south–south partnerships based in the UK, SA and India, working towards shared objectives. Capacity strengthening in this research was informed by the WHO ESSENCE criteria.^11 16^ While this template served its purpose, through the lifetime of the project we were able to recognise the strengths and weaknesses of putting such existing models, which are developed with funders in mind, into practice. ECRs with a range of different professional backgrounds and prior research experience were recruited. For some of the researchers, including those in HICs, this was the first international research collaboration.

**Figure 1:**
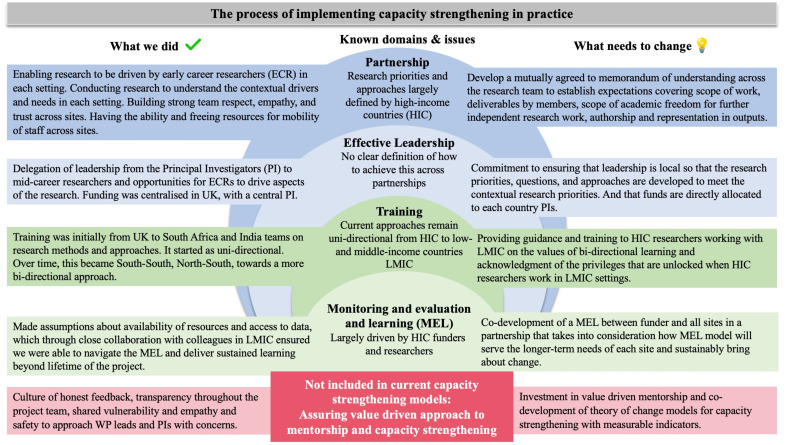
The key features of current capacity strengthening models, using the WHO ESSENCE as a guide, and our lessons on what is currently missing for partnerships across high-income countries (HICs), and low-income and middle-income countries (LMICs). WP, workpackage.

Values that drive academic research are not always aligned with the values one aspires to espouse in human capacity development and equity.^34^ We also learnt that implementing capacity strengthening in practice was shaped by our individual and shared value systems driven by building careers and profile of all team members (figure 1). The need for identifying common value systems across partnerships is rarely considered as part of the initial project planning. Through the process of working on this research collaboration, three key principles to implement human capacity strengthening emerged for us: (1) creating sustainable opportunities for multidirectional partnership and learning, (2) striving for representation in every stage of the research including in academic outputs and (3) committing to developing a legacy and wider impact beyond the research project. These were however driven to a large extend by values of the research team rather than recommended best practice.

Creating sustainable opportunities for multidirectional partnerships and learning through mentorship practices: experiential learning or learning by doing is a model that is recommended in existing capacity strengthening frameworks.^35^ From the onset of the project, sustainable practices to nurture and grow multidirectional partnerships were intentionally built into the life cycle of the project. With a shared interest in mutual and bidirectional learning, early but intentional steps to offset and integrate mentorship as a core value were introduced through induction training, and regular practical project collaboration and support (across settings). We describe two processes from our collaboration to demonstrate what this looked like in practice.

## Induction training

During project initiation, a 2-month on-site extensive induction process was implemented in each study site for the ECRs. Key features of this process included the lead researcher’s support of ECRs through local face-to-face, practical, experiential training and introduction to hospital and surgical teams. Having the same lead researcher work closely with ECRs at both sites enabled consistency in the method and content which was also adapted to suit the cultural setting and context aiding the development of technical skills. An unexpected outcome was how the close collaboration seeded the early start to a culture of openness and transparency in peer review and proved beneficial in the project life cycle when teams, across countries worked on collaborative outputs.

## Cross-country team meetings and peer mentoring and training

Regular meetings between lead researcher and ECRs from India and SA were held at least once a month where ECRs took turns leading and coordinating the meetings. In addition to working through a scheduled agenda together, coaching and mentorship occurred naturally in discussion points. However, initial team building proved challenging across different countries, cultures and contexts, and especially using online platforms. The regularity and consistency of meetings over time, as well as sharing responsibilities and discussing day-to-day current practicalities and challenges, helped to foster a sense of teamwork across the different countries.

Transferring newly acquired skills between the two sites happened through the progression of the project. Cross-country training and sharing skills between ECRs took place. Transfer of research methods skills, including mapping communication using sociogram, was one such example. Training in scoping reviews protocol, literature screening and shortlisting was another example. Over the life of the project, ECRs progressed from two teams to one team working across different sites. While practices were purposefully structured, we could not have anticipated how the maintained connections helped to clarify ongoing project and personal expectations and provided a common and equal platform for professional and self-growth.

The key features of the approach to mentorship, challenges encountered and solutions implemented or recommended are summarised in figure 1. Sustainable benefits of these practices included strengthening individual and research team capacity at each site with project leads and ECRs encouraged to be agents of change at individual sites and across the partnership.^36^ Importantly, the culture of mentorship and allyship extended beyond the ECRs. The principal investigators (PIs) in each site provided a platform for the leadership to be dispersed to enable shared and situational decision-making throughout the project including in dissemination of the research. For a multisite, international project with different substudies, this was an essential component of success.

The strengthening of ECR capacity at both the sites occurred over 3–4 years and resulted in enhancing both hard and soft skills. Hard skills were developed by conducting qualitative research together with data analysis and synthesis, where outputs were published and disseminated through presentations and networking. Soft skills such as leadership and communication skills were supported by formal training,^19–21^ and matured by working closely with surgical teams and managing the research process at the respective study sites. The presence of a highly motivated and investing lead researcher in the team was the key facilitator for this process in addition to the enthusiasm and commitment of the ECRs.

## Striving for representation in every stage of the research including in academic outputs

Existing authorship guidelines often overlook the barriers to ECRs who: (1) lack the practice or training in writing to a Western driven scientific publishing system, and (2) are not under the same pressures to publish or perish that much of global North academia is. These gaps create opportunities for misrepresentation and abuse of power and position in authorship. Working from a value-driven approach recognises the space required for ‘junior’ authors to learn to write to the academic standards of international journals, and expanding authorship to include the team members who significantly contribute to local data collection. In academia, particularly in HICs, often authorship is decided on seniority of academics, and not necessarily their actual contribution to the research.^37–39^ This dispensation is an unwritten rule not captured in ICJME standards. In this collaboration, ECRs were mentored from the onset in research methods, writing and leadership skills. Moving away from extractive research, this approach enabled the ownership of research to remain within country teams to drive the research and have the opportunity to be recognised for their contribution through not only authorship in manuscripts, but also successful research fellowship applications (three), PhD studentship opportunities (two) that were not part of the original funding, and national and international recognition of their work through publications and presentations at scientific events.

## Developing a legacy and wider impact beyond the research project

Creating a sustainable mechanism for bidirectional knowledge exchange and learning enabled individuals (human capacity) to grow so that further research ideas were developed, further grant applications with international collaborations emerged, and there was opportunity for the teams in all sites to expand. Most importantly, it provided a platform for the ECRs in each country to develop their research skills and give them an opportunity to lead research and affect change, for example, being part of the development of the Federation of Clinical Pharmacists in India (https://www.fcpi.in/). This platform also provided opportunities for recognition of clinical pharmacist roles in AMR research in India. One of the PhD studentships that has emerged from this collaboration is investigating the role of clinical pharmacists in AMS programmes across HICs and LMICs. Another has led to nurse-driven interventions across an academic hospital.

The capacity strengthening process in ASPIRES also had its share of challenges, of which the main was COVID-19 pandemic which disrupted the course and timeline of the project resulting in a delay in rolling out interventions and cross-country site visits and face to face trainings. Other challenges included cultural and language barriers which were overcome through regular team meetings and sense checking. Team and research-related challenges were overcome through developing a practice of honest feedback and transparency and adapting to the research environment which was made easy by the constant support of the lead researcher.

## Lessons learnt as part of this process and recommendations for future work

Specific interventions to ensure equity need operational definitions across different domains. The preconditions for this include trust and working from strong relationships developed through existing networks and collaborations. It is important to have conversations at the outset about the different dimensions of success, lay out a plan, review it every 6 months and share progress with the donor as part of progress reports. Modlin *et al* have defined the eight dimensions of equity in global health partnerships specific to clinical trials.^40^ These are: epistemic structures, funding, ethics oversight, regulatory oversight, post-trial access, knowledge translation and research capacity strengthening and maintenance. The challenge is how to create measurable indicators for partnerships to be able to assess progress and effectiveness against specific dimensions for different research collaborations outside of the clinical trials setting. Equally important is to consider the required specific interventions for ensuring equity, the ongoing measures of progress that can be applicable across partnerships for different research and inclusion of indicators of the equity process in progress reports. Based on our collective experience, and the existing evidence, we recommend the checklist in table 1 as a means towards measurable dimensions which can be used by funders, PIs and researchers working in global health and research partnerships.

**Table 1:** Indicator checklist towards equity in multinational research collaborations

Dimension	Checklist items to be considered for each dimension
Funding and resources/budget allocation	We have previously described this in detail,^10^ the summary is below: Were all partners involved in identifying the research gap? Were all partners involved in developing the research questions and epistemological approaches? Are all those individuals who contributed to the research development enabled to be recognised in the leadership, for example, as PI and coinvestigators? Are financial resources being directly given to LMIC institutions with PIs based there? Are resources being allocated equally and commensurate with tasks and activities across the research sites?
Leadership	Is there a MoU that is not only between funder and sites, but across sites and more importantly within the research team (see box 1) Is there a process for shared power across study sites, and within teams? For example, power in leading aspects of work, defining further research questions, power in representing the work externally Are there clear performance management procedures, including probation period, competency checks of staff and managers? Is there a mechanism for conflict resolution across and within teams?
Data equity	Are there mechanisms in place ensuring data ownership and contribution from each partner and not making it just the responsibility of individual sites? Are there mechanisms in place with input from the funder to ensure that the HIC partner is not the only partner with monitoring and oversight and is an active contributor with mutual learning opportunities identified for all partners? Are collaborative visits mutually decided in view of project needs in each site? Do researchers who assisted in data gathering have the opportunity to contribute to the data analysis? Are they provided with adequate training to do so?
Outputs	Is there a dissemination plan in place across and within sites? Is it discussed at the beginning of partnerships? Are all members involved in the discussion and development of the plan? Does the funder have a role in monitoring adherence to the plan?
Capacity strengthening	Is there a baseline assessment of hardware/research infrastructure and human resources capacity of each site? Are there predefined and agreed to goals of what level and type of capacity needs strengthening in the lifetime of the partnership? Is there a strategy for: (1) sustainable opportunities for multidirectional partnership and learning? (2) representation in every stage of the research including in academic outputs? and, (3) committing to developing a legacy and wider capacity strengthening beyond the research project.

HICs, high-income countries; LMICs, low-income and middle-income countries; MoU, Memorandum of Understanding; PIs, principal investigators.

Adopting this checklist is dependent on clear and honest communication which requires self-respect by LMIC PIs/institutions and humility by HIC (often the more powerful) partners. The communication has to be documented and part of MOUs/award agreements because process is equally important and second to ensure HIC partners (or partners with more power) and funders are accountable to some extent.

**Figure 2:**
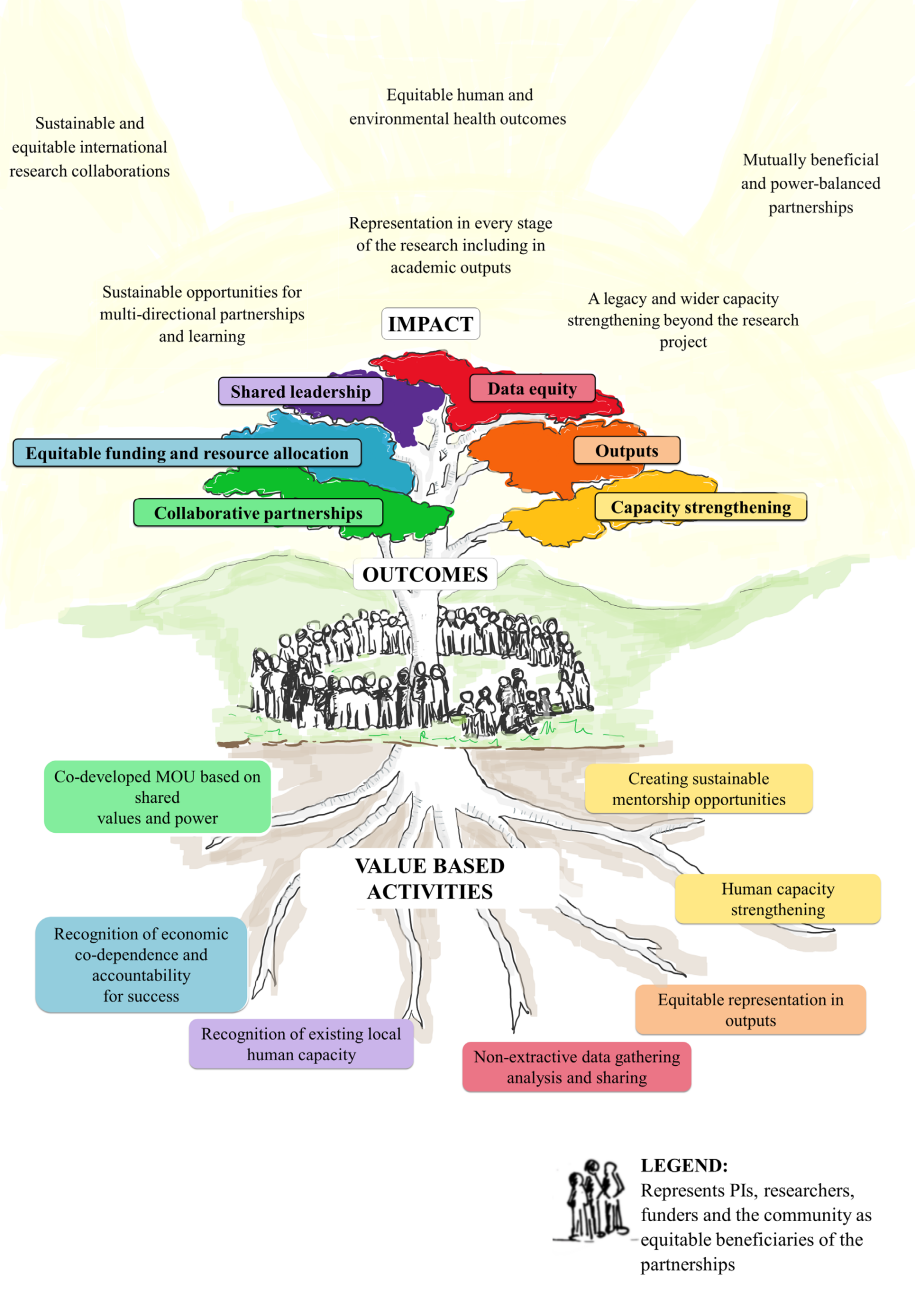
How an equitable partnership driven by value-based activities including mentorship and human capacity strengthening may manifest into achievable outcomes, and impact. MoU, Memorandum of Understanding; PIs, principal investigators.

One of the key lessons learnt through this partnership was to establish mutually agreed to expectations across the team members from the onset of the project and revisit them periodically to ensure continued relevance and validity. We did not establish written and mutually agreed to expectations of work across and within teams in the different countries, beyond generic job descriptions for the research staff and with hindsight we recommend this as an absolute need for successful and productive team work. We have also identified another domain that is not often reflected in capacity strengthening guideline documents that is having a value-driven approach to mentorship and capacity strengthening. We suggest that this is essential for equity within transnational research partnerships as has been previously recommended by other authors,^41^ especially those with a focus on capacity strengthening and in particular human capacity strengthening (figure 2). From our own experience and in discussing this with collaborators, we have developed an outline MoU (box 1) that we suggest needs to be integrated into any research team, particularly teams operating across professions and countries.

Box 1 **An example of a Memorandum of Understanding to be mutually agreed to and periodically reviewed by research team members.**

1. Responsibilities of principal investigators and research leads
  - To define the expectations of deliverables aligned with defined research objectives to the research team.
  - To ensure that research team have access to the training and development opportunities required to deliver to expectations and also to the career development of the individual members of the team.
  - To identify and go through the expected competencies for the job level of research staff with them and identify gaps for training and opportunities within academic institutions for the research skills and career development of the team members. This will also assist setting realistic expectations of pay scale that matches existing competence of team members.
  - To provide opportunities to the team for visibility, mobility across research sites where needed, and recognition of their contribution to the research development and outputs, defining the scope for academic freedom while delivering to the objectives of the funded research.
  - To codevelop with research staff specific milestones and outputs that the team members will be responsible for delivering.
  - To be open to participating in periodic 360 reviews for transparent and constructive feedback on own leadership approach and skills.
  - Where possible and allowed, create within the grant funding budget for team building and leadership development opportunities.
  - Identify a process for conflict resolution across the project within sites and across sites.
2. Responsibilities of research team members
  - To identify their existing competency for the job level they are at and identify gaps for training and opportunities within academic institutions’ competency framework.
  - To proactively with the research leads identify specific milestones and outputs that they will be responsible for delivering.
  - To take responsibility for identifying their training needs to be able to deliver to the specified research.
  - To proactively participate in the research work and contribute to the education, training and development of their peers within their capacity and competence.
  - To be open to participating in periodic 360 reviews for transparent and constructive feedback on own leadership approach and skills.
3. Authorship expectations
  - The discussions on authorship order should take place at beginning of projects, but be reviewed through to ensure those who developed the idea of the research and led on its delivery are duly acknowledged. First and last authorship is dependent on extend of intellectual input, delivery of the work, writing of the manuscript and leadership in entire process. All team members who have contributed to the research need to be acknowledged aligned with the ICMJE authorship guidelines. Where the research is conducted in a specific country(ies) within the collaboration, the authors from those countries need to be fully recognised in authorship order.

It is important to have crucial conversations about expectations in the contribution from perspectives of all team members. Particularly as academic salaries are often not competitive and the nature of academic research that is outputs driven can be considered too demanding across different systems and institutions that do not have the same pressure on PIs.

## Conclusion

In the field of what is currently global health, there is a need to support the leadership and human potential that already exists in LMICs. There also needs to be much greater effort to understand and respond to the existing privileges and disadvantages that arise not from talent and expertise but from geography and circumstances while taking the culture and incentives into consideration. Currently, complex infrastructure and health systems challenges resulting from inequities prevent equitable share of the limited resources in global health research. True transfer of power and equitable partnerships will require the investment of time, intentionality and resources across all sectors. Equitable value-based partnerships in global health research need to be rooted in: (1) codeveloped MoUs based on shared values and power, (2) recognition of economic codependence and accountability for success, (3) recognition of the existing local human capacity, (4) non-extractive data gathering, analysis and sharing, (5) equitable representation in outputs beyond tokenism and (6) human capacity strengthening that includes sustainable mentorship models. Only when we recognise and invest in the human capital across partnerships and acknowledge our codependence can we attain the sustainable and transformative impact that we seek.

## Data Availability

Data sharing not applicable as no datasets generated and/or analysed for this study.
